# Pro-angiogenic Role of Insulin: From Physiology to Pathology

**DOI:** 10.3389/fphys.2017.00204

**Published:** 2017-04-05

**Authors:** Carlos A. Escudero, Kurt Herlitz, Felipe Troncoso, Katherine Guevara, Jesenia Acurio, Claudio Aguayo, Alejandro S. Godoy, Marcelo González

**Affiliations:** ^1^Group of Investigation in Tumor Angiogenesis, Vascular Physiology Laboratory, Basic Sciences Department, Universidad del Bío BíoChillán, Chile; ^2^Group of Research and Innovation in Vascular Health, Department of Basic Sciences, Universidad del Bío-BíoChillán, Chile; ^3^Department of Clinical Biochemistry and Immunology, Faculty of Pharmacy, University of ConcepciónConcepción, Chile; ^4^Department of Physiology, Pontificia Universidad Católica de ChileSantiago, Chile; ^5^Department of Urology, Roswell Park Cancer InstituteBuffalo, NY, USA; ^6^Vascular Physiology Laboratory, Department of Physiology, Faculty of Biological Sciences, Universidad of ConcepciónConcepción, Chile

**Keywords:** insulin, insulin receptor, angiogenesis, endothelial cells

## Abstract

The underlying molecular mechanisms involve in the regulation of the angiogenic process by insulin are not well understood. In this review article, we aim to describe the role of insulin and insulin receptor activation on the control of angiogenesis and how these mechanisms can be deregulated in human diseases. Functional expression of insulin receptors and their signaling pathways has been described on endothelial cells and pericytes, both of the main cells involved in vessel formation and maturation. Consequently, insulin has been shown to regulate endothelial cell migration, proliferation, and *in vitro* tubular structure formation through binding to its receptors and activation of intracellular phosphorylation cascades. Furthermore, insulin-mediated pro-angiogenic state is potentiated by generation of vascular growth factors, such as the vascular endothelial growth factor, produced by endothelial cells. Additionally, diseases such as insulin resistance, obesity, diabetes, and cancer may be associated with the deregulation of insulin-mediated angiogenesis. Despite this knowledge, the underlying molecular mechanisms need to be elucidated in order to provide new insights into the role of insulin on angiogenesis.

## Introduction

Insulin receptor(s) stimulation has pleiotropic effects on endothelial cells (Westermeier et al., 2011). For instance, insulin induces vasorelaxation, enhances endothelial uptake of amino acids (such as L-arginine) and increases survival and migration of endothelial cells (Dubó et al., 2016; Sobrevia et al., 2016). These actions, combined with the capacity of insulin to enhance expression of pro-angiogenic factors such as vascular endothelial growth factor (VEGF), as well as increase pericytes survival and reduce anti-angiogenic protein expression, have established the role of insulin in physiological and pathological angiogenesis (He et al., 2006).

Angiogenesis is the process by which a new blood vessel is formed from a preexisting one (Shibuya, 2013). However, angiogenesis is only one of the mechanisms responsible for vessel formation. Thus, other process such as vasculogenesis (in which angioblast differentiate into endothelial cells leading to de novo vessel formation); intussusception (split of pre-existing vessels); and vascular mimicry (tumor cell line vessels) are also present during vessel formation. In addition, other process such as vessel differentiation into arteries (arteriogenesis) or differentiation of progenitor endothelial cells or bone-marrow-derived cells can occur during revascularization of ischemic tissues. Nevertheless, since vessels nourish nearly every cell in the body, decreased or increased angiogenesis can impact body function. Then, angiogenesis is beneficial for tissue growth and regeneration, but also it can enhance inflammatory response or malignant diseases; or can contribute to cancer metastasis leading to mortality (Cumsille et al., 2015).

Taken the last information into account, it is thought that growth of new blood vessels in the adult -when it is needed- occurs trough vasculogenesis, angiogenesis, and arteriogenesis (Buschmann and Schaper, 1999; Carmeliet, 2000; Semenza, 2007). Therefore, in pathologies, when reduced blood vessel formation is present (such as during wound healing in diabetic patients), those normal stimuli for vessel formation can be down regulated. Contrary, during malignant diseases (such as diabetic retinopathy) up-regulation of those processes is expected. In addition, pro-angiogenic signals can also resemble vasculogenesis in diabetic retinopathy.

In this manuscript, we will review the current information regarding insulin receptor activation, the associated intracellular pathways and the cellular outcomes involved in insulin-mediated angiogenesis. Also in this article, we will highlight the effect of insulin-mediated angiogenesis in pathological conditions such as diabetes, obesity and cancer. Despite the relevance of insulin-like growth factor (IGF-1) and its receptors (IGFRs) to cancer, we have intentionally omitted this topic to focus this review on the role of insulin and insulin receptors on endothelial cells.

## Angiogenesis overview

Angiogenesis is a complex process driven by endothelial and tissue-dependent signals in which at least three sequential steps can be identified: quiescence, activation, and resolution. These processes have been summarized by Carmeliet and Jain (2011) who described that, as a first step in a healthy adult, endothelial cells are quiescent and have a long half-lives. At this stage, endothelial cells are protected against insults by the autocrine action of several maintenance signals including vascular endothelial growth factor (VEGF), angiopoietin-1 (ANG-1), fibroblast growth factors (FGFs), and Notch signaling, as well as by paracrine signals from pericytes, a cell type that cover mature blood vessels. Pericytes also releases pro-survival signals and suppresses endothelium proliferation via VEGF and ANG-1 Therefore, all vessels in a healthy adult exhibit a non-proliferating monolayer of endothelial cells covered by pericytes.

Once pro-angiogenic signals released by subrogating tissue (which include VEGF, ANG-2, FGF, chemokines, inflammatory, and tumor mediators) are sensed for endothelial cells, a series of mechanisms are initiated in order to generate new blood vessels. Initially, pericytes are detached from blood vessel walls and liberate themselves from the basement membrane by proteolytic degradation. Then, endothelial cells loose their junctions leading to dilatation of vessels, increase in the vascular permeability and protein extravasation. Therefore, protein exudates form a provisional extracellular matrix (ECM) scaffold that serve as a platform for endothelial cell migration and formation of nascent vessels (Armulik et al., 2005; Carmeliet and Jain, 2011). To further build a tube and prevent endothelial migration in a chaotic form, a process is required by which one endothelial cells, known as the “tip cells,” becomes selected to lead cell migration in the presence of pro-angiogenic signals, whereas neighbors cells assume subsidiary positions forming “stack cells.” Specialization in tip and stack cells is not a random process, cells are selected according to their levels of VEGF receptors (VEGFRs) expression, in which VEGFR type 2 (VEGFR2) is mainly present in “tip cells,” whereas VEGFR1 is expressed on “stack cells” (Gerhardt et al., 2003; Suchting et al., 2007). In addition, tip cells are equipped with filopodia to sense environmental guidance cues such as ephrins and semaphorins, whereas stalk cells release molecules such as epidermal growth factor-like domain (EGFL7) into the ECM to convey spatial information about the position of the neighbor cells, so that the stalk can elongates. In order to establish a bridge between another vessels branch, other cells types (such as myeloid cells and fibroblast, among other) have a role for establishing a functional communication between neighbor vessels.

**Figure 1:**
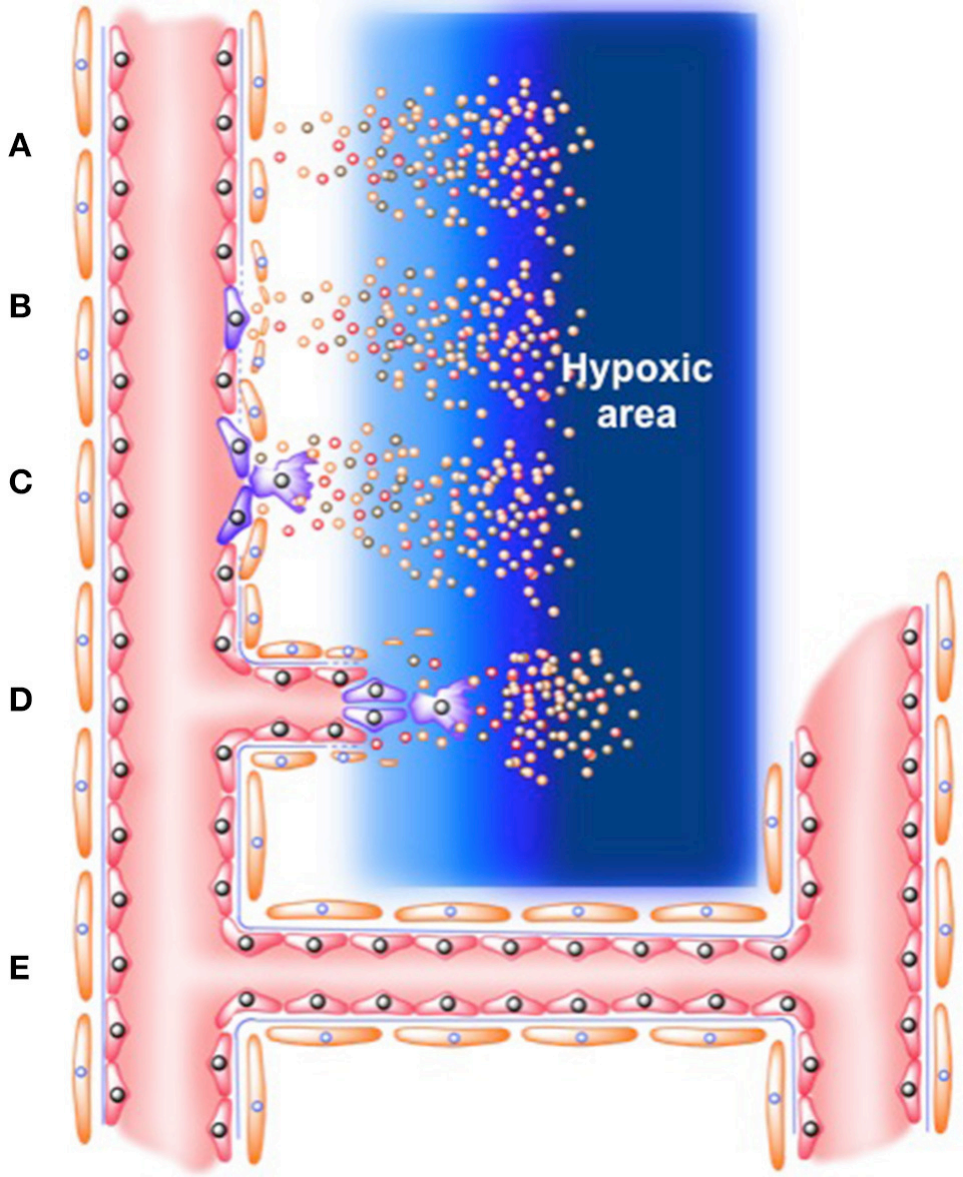
**Role of “tip” and “stack” cells on angiogenesis process. (A)** Release of angiogenic factors (which include VEGF, ANG-2, FGF, chemokines, inflammatory, and tumor mediators) from hypoxic area **(B)**. Activation of endothelial cells in the microcirculation. Mural cells in the vascular wall (i.e., pericytes) are detached, while basement membrane is degraded. Protein exudates form a provisional extracellular matrix scaffold where **(C)**. Tip endothelial cells start to migrate. Meanwhile, neighbor endothelial cells form “stack cells.” **(D)** Formation of nascent new blood vessel. **(E)** Formation of functional vessel as a stable tube in which permanent blood flow is passing through.

The final step in this process includes formation of functional vessels becoming a stable tube in which permanent blood flow is passing through. For endothelial cells to return to their quiescent state and to get covered by pericytes again, several signals are required including platelet-derived growth factor B (PDGF-B), ANG-1, transforming growth factor-β (TGF-β), ephrin-B2, and Notch. Protease inhibitors also participate at this stage-causing establishment of basement membrane; while endothelial cells form new tight junctions to avoid plasma extravasation and ensuring optimal blood flow in the subrogating tissue (Carmeliet and Jain, 2011; Carmeliet and Ruiz de Almodovar, 2013; see Figure 1).

## Insulin and insulin receptors

Insulin is synthesized by the beta cells of the islets of Langerhans. This hormone has 2 dissimilar polypeptide chains, A and B, linked by disulfide bonds. These two chains are derived from a 1-chain precursor, proinsulin. Cytogenetic location of human insulin is located in 11p15.5; which contains 3 exons; exon 2 encodes the signal peptide, the B chain, and part of the C-peptide, while exon 3 encodes the remainder of the C-peptide and the A chain (Steiner and Oyer, 1967). Insulin has far-reaching metabolic consequences including control of circulating levels of glucose, lipids and proteins. Also, circulating levels of insulin are modulated by several hormones and circulating factors such as amino acids, fatty acids, melatonin, estrogen, leptin, growth hormone, among others (Fu et al., 2013). In mammals, insulin also mediates mitogenic effects in a variety of cell types (Stout, 1991; Qiao et al., 2005; Simó et al., 2006; Shrader et al., 2009; Heidegger et al., 2014). However, other reports have failed to find an insulin-mediated endothelial proliferative response (Liu et al., 2009; Lassance et al., 2013).

Differentially to human, in mice and rats, two non-allelic insulin genes are present. Thus, a rodent specific *Ins1* gene that likely arose from the transposition of a reverse-transcribed, partially processed mRNA of the ancestral *Ins2* have been found (Soares et al., 1985). Both genes reside in different chromosomes with differential translational rates and protein function, as showed in *Ins1* or *Ins2* deficient mice (see details in Templeman et al., 2016). For instance, *Ins1*^+/−^:*Ins2*^−/−^ male mice, but not female littermates, were completely protected against diet-induced obesity (Mehran et al., 2012). In a converse genetic manipulation, *Ins1*^−/−^:*Ins2*^+/−^ female mice (Templeman et al., 2015), but not male littermates (Templeman et al., 2016), also prevented obesity. Then, an intricate mechanism of regulation and compensation between each of those genes were present in *Ins1* or *Ins2* deficient male and female mice. Also, *Ins1* and *Ins2* could differentially modulate receptor activity and metabolic consequences of this sex-specific *Ins1/Ins2* effects. These are just examples of how complex is the insulin regulation expression and activity, a fact that is seldom considered in experimental animal model studies.

In human, insulin activates tyrosine kinase receptor expression on the surface of target cells, forming homo- or heterodimers. In general, insulin binds to the insulin receptor (IR), which has at least two isoforms. The first IR isoform lacks exon 11 (exon 11-, IR-A), while the second isoform includes this exon (exon +, IR-B) (see details in Templeman et al., 2016). Insulin can also activate the family of insulin-like growth factor receptors (IGF-IR), which in turn can form homodimers or heterodimers with either IR-A or IR-B (Guzmán-Gutiérrez et al., 2011; Hale and Coward, 2013). Additionally, the IGF-IR exhibits a high level of homology with the IR. Therefore, insulin can activate both families of receptors with different affinities. In this review we will focus on IR-A and IR-B and will provide references to excellent review articles for the study of IGF-IRs (Hale and Coward, 2013).

IR-A is widely expressed throughout the body and is up-regulated during prenatal development and in tumor cells (Gennigens et al., 2006; Belfiore, 2007; Hale and Coward, 2013; Heidegger et al., 2014). In contrast, IR-B is expressed in insulin-sensitive tissues such as the liver, skeletal muscle and adipocytes (Heni et al., 2012). It should be noted that both receptors are expressed on endothelial cells (see details in Hale and Coward, 2013).

The insulin-activated intracellular pathways [Phosphoinositide 3-kinase (PI3K), Mitogen-activated protein kinase (MAPK) and Cbl-associated protein (CAP)] mediate at least three major propagating responses (Westermeier et al., 2011). In the next sections, we will summarize PI3K and MAPK as part of insulin-mediated nitric oxide synthesis. However, insulin also activates GTPase TC10 via lipid-raft localization of the CAP-Cbl-Crk complex and the guanine nucleotide exchange factor C3G, which then initiates glucose uptake (Hale and Coward, 2013).

### Overview of insulin-mediated PI3K signaling pathway activation

The PI3K pathway is one of the most characterized downstream effectors of insulin and activates many of the metabolic functions of this hormone. In brief, binding of insulin to IR triggers its phosphorylation and activation of intrinsic kinase activity, which in turn leads to tyrosine phosphorylation of insulin receptor substrate (IRS) proteins. Phosphorylation of IRS molecules creates Src homology 2 (SH2) binding domains that serve as docking points for SH2-containing proteins like PI3K and the growth factor receptor-bound protein 2 (Grb-2) (see details in Hale and Coward, 2013). The binding of PI3K to IRS-1 activates the catalytic subunit of PI3K, resulting in production of phosphatidylinositol 3, 4, 5-trisphosphate (PIP3). PIP3 promotes phosphorylation and activation of 3-phosphoinositide-dependent protein kinase-1 (PDK-1), which then activates different serine/threonine kinases including Akt (see Muniyappa et al., 2007; Manrique and Sowers, 2014; Manrique et al., 2014). In the vascular field, insulin-mediated Akt activation in endothelial cells leads to enhanced nitric oxide (NO) production via both stimulation of L-arginine uptake and phosphorylation of endothelial NO synthase on Ser^1177^ (activated eNOS) (see Muniyappa et al., 2007; Manrique and Sowers, 2014; Manrique et al., 2014).

### Overview of insulin-mediated MAPK signaling pathway activation

Insulin-mediated activation of the MAPK pathway has been linked with several cell functions including differentiation, proliferation, transformation, survival, and death (Manrique et al., 2014). In brief, this family of proteins consists of members such as P38, extracellular-signal-regulated kinase (ERK), and c-Jun-N-terminal kinase (JNK), with each of them exhibiting a number of different isoforms (Hale and Coward, 2013). The MAPK pathway is activated via a protein phosphorylation cascade that includes Grb2-SOS-Shc-Gab1-SHP2 and Ras (Hale and Coward, 2013). Interestingly, PI3K also binds Ras, producing a cross-talk mechanism between the PI3K and MAPK pathways (Taniguchi et al., 2006; Hale and Coward, 2013). In endothelial cells, insulin-mediated activation of MAPK has been associated with stimulation of L-arginine uptake and NO synthesis (Rodriguez-Viciana et al., 1996).

The insulin-mediated intracellular pathway is far more complex than described in these sections. However, conceptual oversimplification considers two major signaling branches: PI3K-dependent pathways that mediate the metabolic actions of insulin and the MAPK-kinase-dependent pathway that mediates the non-metabolic mitogenic and growth effects of insulin (González et al., 2004, 2011). Furthermore, it has been described that insulin-mediated IR-A activation is associated with a MAPK/AKT (or PI3K) ratio > 1, which has been related to a mitogenic pathway in mouse embryonic fibroblasts. In contrast, insulin-mediated IR-B activation is associated with a MAPK/AKT ratio < 1, which leads to activation of metabolic signaling pathways (Muniyappa et al., 2007).

## General overview of *in vivo* and *in vitro* evidence regarding insulin and angiogenesis

Insulin has pleiotropic effects on the vascular tree and in particular, on endothelial cells. Therefore, it is not surprising that insulin can modulate angiogenesis. Indeed, insulin has been used for nearly 50 years to improve tissue healing in rats (see details in Guzmán-Gutiérrez et al., 2011) and in both diabetic and non-diabetic mice (Gregory, 1965). Additionally, preterm mice lacking insulin receptors in vascular endothelial cells exhibit reduced retinal neovascularization compared to controls (Rosenthal, 1968). Furthermore, diseases associated with altered insulin production and action such as diabetes and obesity exhibit impaired angiogenic processes that are related to microvascular alterations and the pathogenesis of those diseases. We refer to Table 1 and the Section Pathological Implication of Insulin-Mediated Angiogenesis of this manuscript for additional analysis. Briefly, we mentioned that IRs are highly expressed in normal- and cancer-derived endothelial cells (Kondo et al., 2003) and that the activation of these receptors has been related to cell migration, proliferation, endothelium survival, and VEGF expression during tumor angiogenesis. Additionally, insulin receptors are expressed in pericytes (contractile cells that wrap around the endothelial cells) in which they modulate endothelium-pericyte interaction, a fundamental step for vessel maturation (Liu et al., 2009; Rensing et al., 2010; Westermeier et al., 2011).

**Table 1:** **Pathological vascularization in diseases related to insulin alterations**.

	**Vascularization defects**	**References**
Diabetes	Reduced vascularization	Escudero et al., 2014
mellitus	• Impaired wound heling	Martin et al., 2003
	• Impaired recovery after cardiac infarction	Liu et al., 2009; Kolluru et al., 2012; Hrynyk and Neufeld, 2014
	• Embryonic vasculopathy	Chou et al., 2002; He et al., 2006
	• Transplant rejection	Hattersley and Tooke, 1999
	Hypervascularization	
	• Diabetic retinopathy	Zhang et al., 2004; Cheng et al., 2007
	• Diabetic nephropathy	Poulaki et al., 2002; Simó et al., 2006; Kobayashi and Puro, 2007; Meng et al., 2012
	• Increased risk for cancer Breast, colon, prostate, kidney, pancreas	Chiarelli et al., 2000; Baelde et al., 2004
Gestational diabetes	Placental hypervascularization	Belfiore, 2007; Giovannucci et al., 2010
Obesity	Hypervascularization of adipose tissue	Escudero et al., 2013; Lassance et al., 2013
Cancer	Increased risk for prostate cancer	Jung et al., 2012; Cao, 2013

Insulin analogs such as arg-insulin exhibit high pro-proliferative activity in human osteosarcoma cells with a potency at least 8 times greater than human insulin (Hale and Coward, 2013). Additionally, insulin resistance and hyperinsulinemia may promote tumor angiogenesis and tumor development directly through activation of insulin receptors or indirectly by increasing the levels of insulin-like growth factors (IGF), steroid sex hormones, inflammatory processes, and by disrupting adipokine homeostasis (Kurtzhals et al., 2000). Insulin and insulin analogs also show high capacity to stimulate angiogenesis *in vitro* (Jalving et al., 2010). In contrast, metformin, an insulin-sensitizing drug, reverts increased levels of VEGF, angiopoietin-like protein (ANGPTL 1), and the ANGPTL 1/ANGPTL 2 ratio, as well as decreased levels of platelet-derived growth factor B (PDGFB) and platelet-derived growth factor D (PDGFD) observed in hyperinsulinemic animals (Rensing et al., 2010). Therefore, metformin may have anti-angiogenic properties (Di Pietro et al., 2015).

Insulin, directly or indirectly, has been linked with angiogenic processes. In the next sections, we will discuss evidence that supports a direct pro-angiogenic role of insulin on endothelial cells, on the synthesis and release of pro-angiogenic factors, and on endothelial-pericyte interactions.

## Insulin and pericytes

Pericytes are a class of mural cells that provide support to blood vessels of all sizes. Conventionally, they have been described as a component of smaller vessels such as capillaries, arterioles, and venules. Although pericytes were originally thought to act as support cells, they actually participate in vessel contraction by regulating vascular diameter and capillary blood flow. Pericytes also control endothelial membrane folding and luminal cytoplasmic protrusions. These cells establish active cross-talk with endothelial cells that promote endothelial cell differentiation and maturation (see details in Viollet et al., 2012). Pericytes also have been linked with the maintenance of endothelial survival and the inhibition of endothelial cell migration and sprouting processes through VEGF expression and also serve as multilineage progenitor cells (see details in Richards et al., 2010). Therefore, pericytes normally play a role in adjusting the angiogenic process, and pericyte loss or dysfunction has been noted in the earliest phase of diabetic retinopathy (see details in Richards et al., 2010).

The initial description of IR expression on human pericytes was reported in the 80s (Kobayashi and Puro, 2007). Using ^125^I-radiolabelled insulin and binding experiments, high affinity receptors, similar to those identified on retinal endothelial cells, were characterized on pericytes (James and Cotlier, 1983). Interestingly, insulin was more effective for enhancing cell proliferation of pericytes than endothelial cells (King et al., 1983). More recent evidence have shown that insulin treatment for glycaemia reduction in diabetic patients was associated with an elevated number of circulating pericyte progenitor cells after 3 months of treatment (King et al., 1983). Therefore, IRs are present in pericytes and appear to enhance pericyte proliferation and mobilization of pericyte progenitor cells from bone marrow.

The functionality of IRs on pericytes has been studied using cells isolated from bovine retinal capillaries, in which insulin induced a slow hyperpolarization in a dose-dependent manner (Fadini et al., 2012). Further characterization analyses revealed the participation of both Kir and small-conductance Ca^2+^-sensitive potassium channels in the insulin-induced hyperpolarization of these cells. Kobayashi and Puro (Berweck et al., 1993) used normal rat retina to show that insulin-reduced endothelial cell death occurs only in microvessels that contain pericytes, an effect that was associated with PI3K and ERK activation.

**Figure 2:**
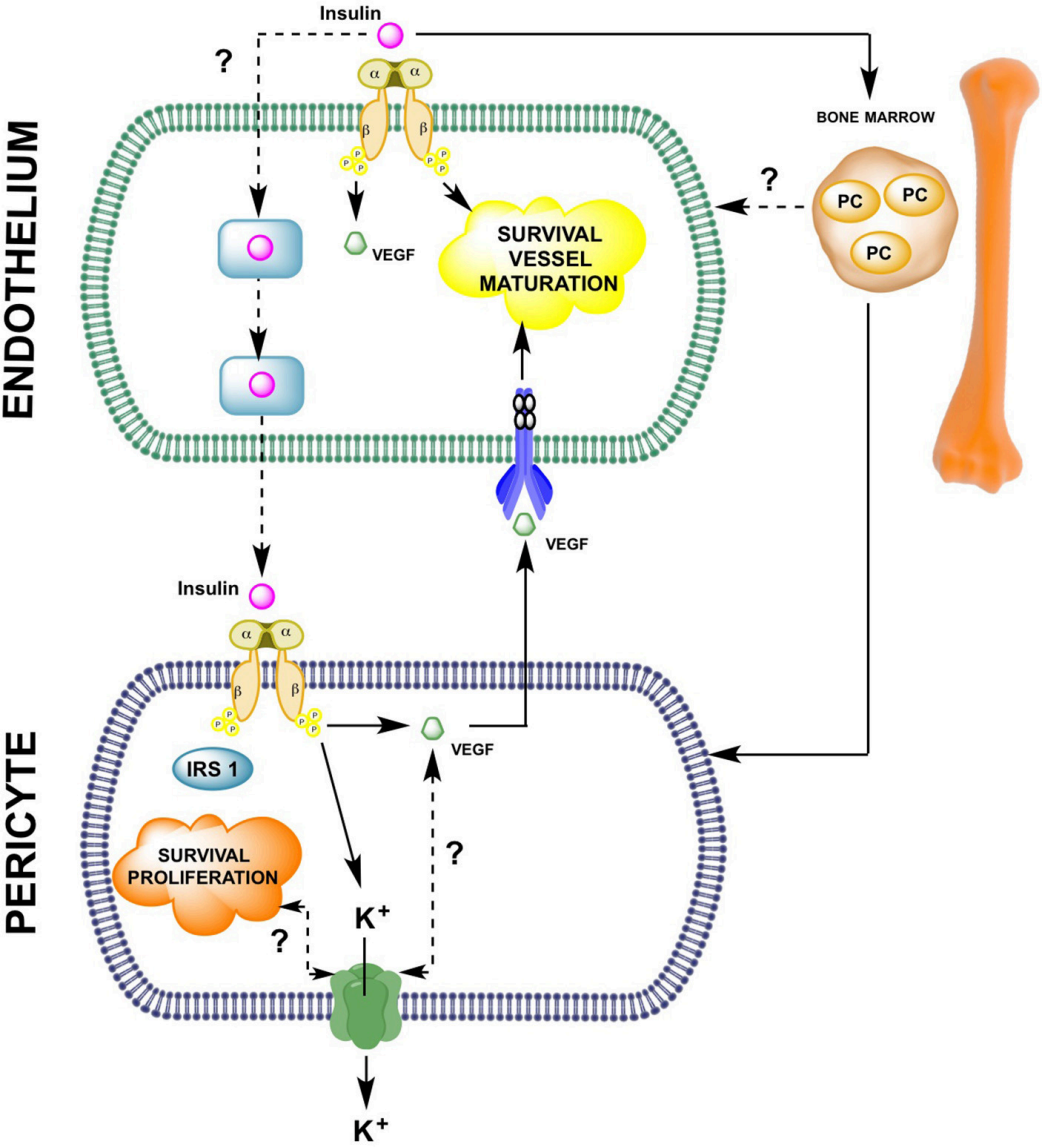
**Insulin-mediated pericyte and endothelial cell-to-cell interaction during angiogenesis**. As described in this manuscript, few reports have analyzed the potential role of insulin in the endothelial-pericyte interactions during cell proliferation/migration or vessel maturation. The molecular mechanisms summarized in this schematic representation indicate that on endothelial cells, insulin, either directly or via vascular endothelial growth factor (VEGF) expression, is involved in promoting angiogenesis. Insulin also activates insulin receptors present on pericytes leading to survival and proliferation of these cells. In pericytes, insulin induces expression of VEGF, which might reach its respective receptors on the endothelial cell surface and control endothelial survival. Alternatively, insulin can activate mobilization of progenitor cells (PC), which might differentiate into either pericytes or endothelial cells. Until now it is unknown whether insulin uses transcellular or paracellular transport mechanisms in endothelial cells to reach the subendothelial space and then activate insulin receptors on pericytes.

Despite limited information about the direct role of insulin on pericyte function during angiogenesis, it has been shown that insulin protects human brain pericytes from the toxic effect of beta amyloid mimic Abeta1-40 in a dose-dependent manner (2007). Thus, insulin could protect cerebral tissue from damage during Alzheimer disease by inducing pericyte survival and vascular recovery. More studies are needed to clarify the significance of insulin effects on pericyte-endothelium interaction or angiogenesis (see Figure 2).

## Insulin and endothelium

**Figure 3:**
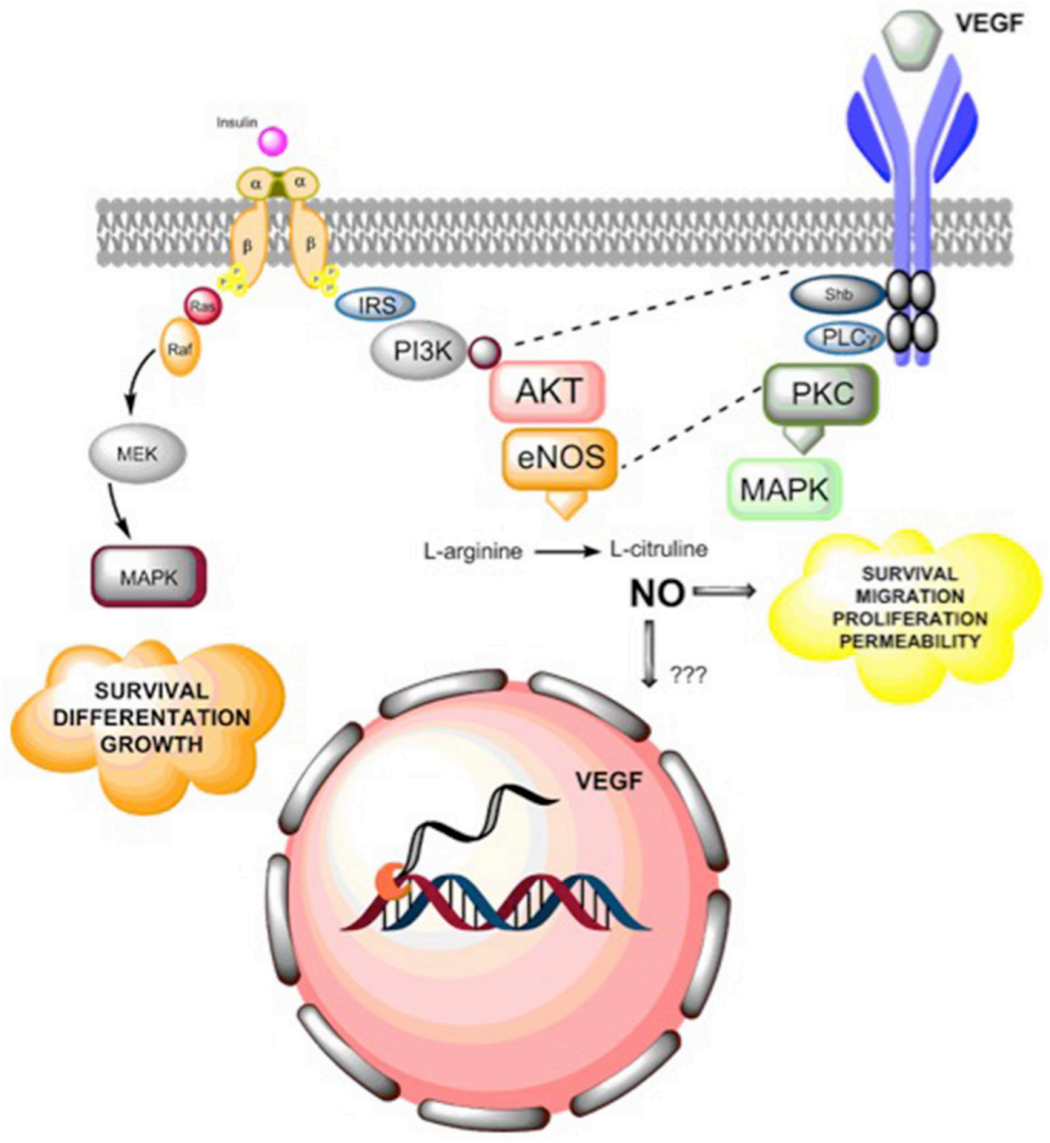
**Summary of insulin actions in angiogenesis**. Insulin receptor activation leads to endothelial cell survival, differentiation and growth via activation of MAPKs. Additionally, insulin receptor activation leads to nitric oxide (NO) synthesis via PI3K-AKT activation, which in turn regulates endothelial survival, migration, proliferation, and vascular permeability. Additionally, NO may regulate the expression of vascular endothelial growth factor (VEGF), but the molecular mechanisms that underlie this effect are still unclear. Alternatively, insulin mediates a pro-angiogenic effect by expression and secretion of VEGF, which then can activate VEGF receptors (mainly VEGFR2), leading to activation of phosphorylation cascades including cross-talk (dashed lines) between insulin-mediated PI3K and endothelial nitric oxide synthase (eNOS) or by directly activating MAPKs generation of pro-angiogenic effects on endothelial cells.

Studies of angiogenesis *in vitro* have examined cell proliferation, cell migration, tube formation capacity, expression and/or activation of pro-angiogenic factors such as VEGF and VEGF receptor (VEGFR). Effects of insulin on these parameters are summarized in Figure 3.

Insulin increased endothelial cell migration in a manner that was not blocked by VEGFR type 2 (VEGFR2) inhibitor SU1498 (Rensink et al., 2004). Further characterization of that phenomenon showed that insulin-mediated migration required participation of PI3K, Akt-sterol regulatory element-binding protein-1 (SREBP-1) and Ras-related C3 botulinum toxin substrate 1 (Rac-1) (Liu et al., 2009). Afterward, insulin could induce a VEGF-independent mechanism involving activation of the PI3K-Akt-SREBP-1-Rac1 pathway, leading to cell migration.

In terms of tube formation or *in vitro* angiogenesis, Lassance et al. (2013) showed that insulin enhanced *in vitro* angiogenesis and caused actin reorganization. This effect was associated with phosphorylation of IR and IRS-1, as well as Akt, glycogen-synthase kinase-β3 and endothelial NOS (eNOS). This in turn caused increased nitric oxide (NO) production and activation of Rac1.

Insulin also stimulated cell proliferation in several cell lines or primary cultures including arterial smooth muscle, fibroblasts, and epithelial cells (Lassance et al., 2013). However, the effect of insulin on endothelial cell proliferation is still controversial. Several studies reported an increase in proliferation (Stout, 1991; Jiang et al., 2003), while other studies reported no effect (King et al., 1983; Yamagishi et al., 1999; Liu et al., 2009; Shrader et al., 2009) compared to untreated cells. In this regard, insulin increased the area occupied by CD31+ or endothelial cells and the formation of mature blood vessels (identified by pericyte coverage) in a rat model of subcutaneous healing (Nagai et al., 2003; Liu et al., 2009). King et al. (1983) also showed that microvascular endothelium isolated from calf retina exhibited a dose-dependent increase in cell proliferation in the presence of insulin (1–1,000 nM), whereas endothelium derived from calf aorta showed no such effect. This differential response was observed only with insulin because other stimuli such as serum or endothelial growth factors induced similar proliferative responses in both endothelial cell types. The differences can be attributed to the fact that micro- and macro-vascular endothelial cells can respond to insulin differentially (1983).

## Insulin and VEGF

The proliferative response mediated by insulin has been associated with higher VEGF levels in some endothelial cell types. For instance, insulin-induced proliferation of human umbilical vein endothelial cells (HUVEC) was associated with activation of PI3K, Akt, and up-regulated VEGF protein expression (King et al., 1983; Lang et al., 2003; Sobrevia et al., 2011). The latter effect was confirmed by other reports involving human microvascular endothelial cells in which insulin increased VEGF mRNA levels (Shrader et al., 2009) or extracellular protein levels of VEGF (Yamagishi et al., 1999).

The underlying pathway for insulin-mediated expression of VEGF depends on several conditions including cell type, time and doses of incubation. Thus, insulin increased VEGF expression via p38 mitogen-activated protein kinase (MAPK) and PI3K, but not via p42/p44 MAPK or PKC pathways in retina of diabetic rats (Liu et al., 2009). Jiang et al. (2003) confirmed that insulin enhanced VEGF mRNA levels in a time- and dose-dependent manner with biphasic behavior in aortic smooth muscle cells. Accordingly, in this model, a rapid phase (100 nM, 1 h) for insulin-mediated VEGF expression depended solely on PI3K activation, whereas a second phase (12 h) depended on both PI3K and p42/p44 MAPK pathways. Nevertheless, in cardiomyocytes isolated from neonates and adult rats, it has been shown that insulin increased VEGF mRNA and protein expression in the culture medium in a dose-dependent manner (0–250 nM). To increase the levels of VEGF expression, insulin requires the PI3K/Akt pathway, but not the Ras/MEC/Erk-1/2 pathway in this cell type. Further characterization in cardiomyocytes found that insulin-mediated VEGF expression requires Akt2, but not Akt1 (2003).

*In vivo* experiments also have confirmed insulin-mediated expression of VEGF. Insulin was found to enhance VEGF and VEGFR1 mRNA levels without changes in VEGFR2 mRNA levels in a rat model of hepatic regeneration after partial hepatectomy (He et al., 2006). In the same model, protein levels of VEGF also increased at 72 h after insulin treatment (Qiao et al., 2005). In streptozotocin-induced diabetic rats, the level of VEGF mRNA expression was increased, whereas the VEGFR1 mRNA level was unaltered in aortas from insulin-treated diabetics (5–30 U/Kg/day for 1 week) compared to either untreated diabetics or non-diabetics treated with insulin (Qiao et al., 2005).

A more direct analysis regarding the physiological significance of IRs and their signaling pathways in VEGF expression and vascularization was performed using hearts from muscle-specific insulin receptor knockout (MIRKO) mice. In these studies, insulin injection (5 U/Kg) in MIRKO animals reduced Akt activation, VEGF mRNA levels, and expression of the endothelial cell marker, VE-Cadherin (Kobayashi and Kamata, 2002). Furthermore, in this study, the authors evaluated the level of vascularization in the peri-infarct zone from ventricular tissues of MIRKO and control mice at 7 days after coronary artery ligation. Their results showed a high mortality rate in MIRKO animals, which was associated with reduced blood vessels in the peri-infarcted zone without changes in endothelial cell proliferation. Therefore, these results showed that the insulin-PI3K/Akt2-VEGF pathway is a key factor in the recovery of vascularization after myocardial ischemia.

### Insulin and placental growth factor

The placental growth factor (PLGF), which belongs to the family of VEGF factors, has also been associated with angiogenesis. In this regard, ARPE-19 cells exposed to hypoxic conditions or to insulin induced an up-regulation of PLGF expression (He et al., 2006). Considering this effect, insulin, via PLGF up-regulation, might control endothelial cell permeability, which in turn activates the PLGF-mediated ERK signaling pathway. These effects resulted in the opening of the retinal pigmental endothelium tight junctions, and consequently, have been involved in retinal edema (Miyamoto et al., 2007).

## Insulin and angiopoietins

Angiopoietin (Ang) and Tie receptor tyrosine kinases (Tie) constitute another family of proteins that are almost exclusively expressed in endothelial cells (Miyamoto et al., 2007). In humans, Ang1, Ang2, and Ang4, which bind Tie1 and Tie2 receptors, constitute this family of proteins. The activation of these receptors has been linked to vessel quiescence in adults, as well as the regulation of vascular remodeling and maturation of blood and lymphatic vasculature during later phases of embryonic and postnatal development (Eklund and Saharinen, 2013). Ang1 functions as a Tie-2 agonist, whereas Ang2 is a natural inhibitor of the Tie-2 receptor that antagonizes the Ang1-specific vascular protective function (Eklund and Saharinen, 2013). Because the ligand for Tie-1 has not been identified, this orphan receptor may act as a negative regulator of Tie-2; however, its precise role is still unclear (Rasul et al., 2012). Additionally, the activation of Tie-2 receptors by Ang1 drives a negative-feedback loop for Ang2 production on endothelial cells (Augustin et al., 2009; Carmeliet and Jain, 2011); therefore, Ang-Tie is a highly self-controlled system.

Very little is known about the interaction between insulin and angiopoietins. However, the relationship between these two main regulators is indirectly manifested in patients with type 2 diabetes mellitus (T2DM). Previous observations, including a large third-generation cohort of the Framingham Heart Study, showed a positive association between circulating Ang-2 levels and the occurrence of diabetes, but an inverse relationship with total cholesterol and diastolic blood pressure (Daly et al., 2004). Meanwhile, soluble Tie-2 was positively associated with body mass index and triglycerides (Lieb et al., 2010). Unfortunately, no analyses of insulin administration were performed in this study.

Additionally, in a case-control study, Li et al. (2015) found that serum Ang-2 levels present in T2DM patients with angiopathy were significantly higher compared to patients without angiopathy (Lieb et al., 2010). Furthermore, Ang-2, but not Ang-1, levels were positively correlated with a homeostasis model assessment for insulin resistance (HOMA-IR) and glycosylated hemoglobin A1c (HbA1c). This indicates that Ang-2 may constitute a marker of dysfunctional angiogenic process in these patients.

On the other hand, angiopoietin-like proteins (ANGPTL) are orphan ligands (at least 7 members) that have similar structure domains to angiopoietins but do not bind to Tie-1 or Tie-2 receptors. From these proteins, ANGPTL-1-4 and ANGPTL-6 are functionally expressed in endothelial cells and control survival and migration-inducing signals leading to angiogenesis. To the contrary of what was proposed for Ang1, ANGPTLs are synthesized not only in endothelial cells but also in many other cell types. Furthermore, ANGPTL2, 3, 4, and 6 molecules can be detected in the circulation (Li et al., 2015). Additionally, Tie-2-mediated activation of PI3K has been associated with cell migration (see details in Augustin et al., 2009), three-dimensional capillary organization, proliferation, and increased expression of basement-membrane-degrading proteases (Master et al., 2001). Also, ANGPTLs may regulate lipid, glucose and energy metabolism (see details in Augustin et al., 2009). However, as far as we know, there is no information regarding the potential role of insulin on ANGPTL-mediated angiogenesis.

## Pathological implication of insulin-mediated angiogenesis

### Diabetes type 1 and type 2 and angiogenesis

Since type 1 (T1DM) and type 2 (T2DM) diabetes are pathophysiologically different diseases, it is expected that vascular complication will also be different. For instance macroangiopathy occurs more frequently in type 2 diabetes, while microangiopathy is a common feature of both types of diabetes. In terms of angiogenesis, in T1DM and T2DM, there are two tissue-specific paradoxical changes in small blood vessels in diabetes (Simons, 2005). One is an excessive, and uncontrolled formation of premature blood vessels in some tissues such as the retina, in which neovascularization is a hallmark of late-stage diabetic retinopathy and atherosclerotic plaque destabilization. The other is the deficiency in the formation of small blood vessels in peripheral tissues such as the skin, which contributes to the impaired wound healing in the skin, fibrosis and deficiency in collateral vessel development. Therefore, in diabetes, angiogenesis can be up regulated or down regulated, depending of duration of disease and compliance in the metabolic control (Nentwich and Ulbig, 2015).

Regarding differences in angiogenesis in T1DM and T2DM, Yan et al. (2009) evaluated postischemic neovascularization in streptozotocin-treated (type 1 diabetes model); type 2 diabetic Lepr(db/db) and control mice. They found that postischemia recovery of hind limb perfusion was less in T2DM than T1DM mice. Also, they found a reduced endothelial progenitor cell incorporation into tubular structures in T2DM mice. They concluded that T2DM mice displayed a significantly less effective angiogenic response to ischemia than T1DM mice.

Preclinical studies also have found an impaired VEGF-induced migration in marrow-derived EPCs from T1DM and T2DM, as well as impaired incorporation of EPCs into tubular structures that was less effective in T2DM than T1DM rodents (Yan et al., 2009). These results suggest an impairment of EPC mobilization and function; therefore processes such as vasculogenesis and/or endothelial repair might be differentially affected in T1DM and T2DM.

In human, previous results also suggest a differential angiogenesis process in T1DM and T2DM patients. For instance, VEGF-A was most intensely present in vitreous samples and neovascular tufts from T1DM patients with proliferative retinopathy. While, VEGF-D was more abundant in T2DM than T1DM patients (Kinnunen et al., 2009). Also, differential sequence variations in the *VEGFA* gene were associated with elevated risk for developing blinding diabetic retinopathy in T1DM and T2DM patients (Abhary et al., 2009). Thus, after controlling for sex, HbA1c, and duration of disease, in T1DM, the AA genotype of rs699946 and the GG genotype of rs833068 were most significantly associated; while in T2DM, the C allele of rs3025021 and the G allele of rs10434 were most significantly associated with blinding diabetic retinopaty. How these differences may impact the pathophysiology of diabetic retinopathy or angiogenesis process in general are still unknown.

#### Microvascular dysfunction in the diabetic retina

According to the evolution of the clinical diabetes, after 20 years of having this disease, almost all T1DM patients, 80% of insulin-dependent diabetes patients, and 50% of insulin-independent T2DM patients will have vision loss due to diabetic retinopathy (Chou et al., 2002). Globally, the prevalence of diabetic retinopathy is estimated to be 126 millions of the 382 million people with diabetes (Zheng et al., 2012). Clinically, diabetic retinopathy has two forms: nonproliferative diabetic retinopathy (NPDR) and proliferative diabetic retinopathy (PDR), which in turn is related to absence or presence of neovascularization, respectively.

The pathophysiology of diabetic retinopathy (DR) is complex and multifactorial. Endothelial dysfunction has been recognized as a key factor and includes capillary basement membrane thickening, damage of vascular wall structure with endothelial cell and pericytes loss associated with impairment of blood-retinal barrier, and increased neovascularization (mainly due to angiogenesis) (Dhoot and Avery, 2016). Thus, diabetic patients exhibit an enhanced angiogenic process that contributes to DR, nephropathy and atherosclerotic plaque destabilization (Martin et al., 2003). Additionally, epidemiological studies have shown that insulin therapy is an independent risk factor for the progression of intraocular neovascularization in diabetic retinopathy (Martin et al., 2003). Therefore, angiogenesis is a key factor in the generation and/or progression of vascular alterations in diabetes.

In terms of neovascularization, dysfunction of endothelial progenitor cells (EPC) has been reported in patients with T1DM and T2DM (see details in Lois et al., 2014). However, determination of the number of circulating EPC in diabetic patients has evidenced conflicting results, not only considering T1DM or T2DM, but also according to severity of the disease. We would like to refer the excellent review by Lois and colleagues (see details in Lois et al., 2014).

Multiple studies have identified VEGF as a key factor in the pathogenesis of DR in particular PDR (Yu et al., 2016). For instance, a positive correlation among patients with poor glycemic control and increased VEGF plasma levels has been found (Zehetner et al., 2013), while high VEGF levels were found in aqueous and vitreous with PDR in comparison with NPDR (Dhoot and Avery, 2016; Mohamed et al., 2016). In accordance with this mechanism, anti VEGF therapy has been tested in clinical trials in a small number of PDR patients with some promissory effects (Dhoot and Avery, 2016; Mohamed et al., 2016).

#### Diabetes, healing, and angiogenesis

Diabetes mellitus is a disease associated with impaired neovascularization that leads to reduced revascularization of ischemic tissues; impaired wound healing; embryonic vasculopathy and organ transplant rejection in diabetic patients (Oike et al., 2005). Impaired wound healing is also referred to as chronic wound including slow healing and non-healing wounds. Re-epithelialization and angiogenesis are two essential steps in wound healing. Since, wound healing is delayed in the presence of high levels angiogenic inhibitors, and promoted by local administration of VEGF (Barrientos et al., 2014), it is not surprising that this marker has been linked to impaired wound healing process observed in diabetic patients. Indeed, recombinant VEGF applied topically to chronic diabetic neuropathic foot ulcer, three times a week for up to 6 week, reduced in at least 10 days the ulcer healing in diabetic patients (Hanft et al., 2008).

Nonetheless, little information has been found regarding the angiogenic role of insulin in diabetic subjects. For instance, insulin administration enhanced VEGF in cardiomyocytes isolated from diabetic rats (Simó et al., 2006) and reduced circulating anti-angiogenic proteins (angiostatin and endostatin) in Yucatan miniswine (Chou et al., 2002). In humans, diabetes and insulin resistance have been associated with reduced VEGF expression in the heart, which may decrease capillary density in the myocardium of diabetic and insulin-resistant patients (Boodhwani et al., 2007). Insulin use has also been associated with some specific cancers (see below). However, more information is required to better understand the impact of insulin treatment in diabetic patients.

### Cancer, diabetes, and insulin-mediated angiogenesis

Insulin as a growth factor has been linked with tumor growth in prostate (Aiello et al., 1994) and breast cancers (Heni et al., 2012), among others. Epidemiological evidence has also shown that patients with T2DM have increased risk for breast, colon, prostate, kidney, and pancreatic cancers (Belfiore, 2007; Huang et al., 2011; Kalla Singh et al., 2011), an effect that has been associated with high insulin plasma levels (Giovannucci et al., 2010). Accordingly, T2DM patients who used insulin analogs such as glargine exhibited a high risk of cancer in a dose-dependent manner (adjusted hazard ratio was 1.1, 1.2, and 1.3 for daily doses of 10, 30, and 50 U, respectively) (Jalving et al., 2010). On the other hand, T2DM patients who were treated with metformin, an insulin sensitizer, showed a reduced risk of cancer (odds-ratio for any exposure to metformin was 0.79) (Hemkens et al., 2009; Grouven et al., 2010; Rensing et al., 2010). Additionally, Evans et al. (2005) and Jalving et al. (2010) found an inverse dose-response relationship between duration of exposure to metformin and cancer incidence. Also, reduced incidence of neoplastic diseases and cancer mortality in T2DM patients treated with metformin has been reported (2005). Interestingly, reduction in the relative risk seems to be specific to certain cancers such as prostate, pancreas and breast cancers (see Table 1).

The mechanisms for the angiogenic role of insulin-sensitizing drugs on cancer development are not well understood. However, the role of IRs seems to be noticeable. On this regard, overexpression of IR-A, but not IR-B, has been linked to increased proliferation and migration in prostate cancer and non-cancer cell lines (see details in Viollet et al., 2012). Interestingly, using chorioallantoid membrane assays, Heidegger et al. (2014) have shown that a prostate cancer cell line (PC3) overexpressing IR-A and seeded on chorioallantoid membranes exhibited higher tumor growth compared to control. Complementarily, tumor vascularization was enhanced in tumors generated by cancer cells overexpressing IR-A. However, this effect was reduced in cancer cells in which IR-B expression was down regulated. This effect indicates participation of both receptors in angiogenesis of prostate cancer.

### Obesity, insulin, and angiogenesis

Preadipocytes have endothelial and perivascular origins, suggesting that adipogenesis, angiogenesis, and vascular remodeling are tightly and coordinately regulated (Heidegger et al., 2014). Additionally, the histological appearance of adipose tissue remains a “honeycomb-like” structure in which each adipocyte is embedded in a vascular chamber. On the other hand, adipose tissue expansion was associated with compensatory increased vasculature that resembles tumor growth. Interestingly, this phenomenon is related to insulin resistance (Cao, 2013). Except for the findings of these studies, the role of the vascular network on adipocyte function is not completely understood. Indirect evidence shows that decreased blood supply leads to hypoxia and inflammation in obese people. This exacerbates insulin resistance (Cao, 2013) but can also stimulate angiogenesis (Cao, 2013). Adipose angiogenesis reduces adipose tissue hypoxia and fibrosis. Therefore, it is expected that more vascular endothelial cells in adipose tissue can mitigate the effects of insulin resistance in obese or diabetic patients (Ahmed et al., 2000). However, how insulin resistance generated during obesity might impact adiposity-mediated angiogenesis remains unclear.

Classically, it has been identified the muscles, liver, and adipose tissue as the major targets for insulin resistance. Also, vascular cells, and in particular endothelial cells are selectively affected by insulin resistance. This is important, since endothelial monolayer is the first cell type encountered during insulin delivery and may contribute to tissue specific affection of angiogesis. Regarding adipose tissue, visceral adipose tissue (VAT) is more closely linked to insulin resistance than subcutaneous adipose tissue (SAT). Thus, they are metabolically different, and angiogenesis could be differentially affected in those tissues. In this regards, near to 50% of the adipose tissue, particularly VAT, secretome was composed of factors with a role in angiogenesis (Hocking et al., 2010). However, as far as we known, literature lack of information about tissue specific affection of angiogenesis in the frame of insulin resistance.

## Concluding remarks and future directions

Insulin, as a hormone involved in tissue growth and recovery after injury, is involved in the control of angiogenesis through at least four mechanisms reviewed in this article: (1) control of the interaction between endothelium and pericytes; (2) endothelial cell migration and proliferation (particularly in microcirculation); (3) synthesis of pro-angiogenic factors such as VEGF and Ang; and (4) regulation of tissue metabolism, which indirectly affects endothelial cell survival. Despite the fact that these effects are well described in the literature, the underling signaling pathways are not entirely known.

In particular, functional expression of IR-A and IR-B have been described on endothelial cells and pericytes, which trigger intracellular signaling involving PI3K and MAPK pathways, as well as subsequent activation of phosphorylation cascades that lead to synthesis of pro-angiogenic factors such as VEGF and Ang. These mechanisms result in modulation of cell migration, cell proliferation, *in vitro* angiogenesis, endothelial differentiation and survival. Because angiogenesis involves all of these effects, the general agreement is that insulin is a pro-angiogenic hormone. It is not clear, however, whether all of these effects are triggered in all endothelial or vascular cells.

These mechanisms become uncertain when considering, for example, which type of insulin is being used (natural or pharmaceutical preparation), or whether insulin-sensitizing drugs are used. Further complexity should be analyzed considering which IRs are present in any specific endothelial cell type (macrovascular or microvascular endothelium). On this regard, future analyses should consider that not only IRs, but also VEGFRs, are differentially expressed on endothelial cells. More specifically, future investigations should consider which specific tyrosine is phosphorylated during VEGFR2 activation, because differential cell responses can be triggered accordingly (Jung et al., 2012). Moreover, future studies should be designed considering that intracellular pathways associated with either insulin or VEGF exhibit active cross-talk inside the cells. Lastly, we could not rule out the participation of IGFs and IGFRs in many, if not all, of the angiogenic mechanisms discussed in this article.

Pathological implications of these mechanisms in T2DM, cancer or obesity are even less known. We highlighted in this article the case of T2DM or insulin resistance, in which a reduction of insulin activity led to microvascular alterations (in skin, eye, kidney, and neurovascular tissues, among others). It also seems to be evident that recovery of insulin effects by either pharmacological use of insulin or by using insulin-sensitizers would help to prevent the occurrence of microvascular alterations in diabetes. However, epidemiological analyses have shown that T2DM patients under glycemic control, in particular, those who use insulin analogs, have at least a 30% higher risk for developing cancer in breast, colon, prostate, kidney and pancreas tissues. Clinical and basic scientists should be aware of this information and conduct future investigations to clarify the mechanisms behind the relationship between cancer risk and use of insulin analogs.

## Author contributions

This work was conducted as a collaborative effort among all the cited authors. CE defined the research topic. KH, FT, KG, and JA prepared the draft of the manuscript. AG, CA, MG, and CE edited the text. All authors approved the final version of the manuscript.

### Conflict of interest statement

The authors declare that the research was conducted in the absence of any commercial or financial relationships that could be construed as a potential conflict of interest.

## Abbreviations

PDK-1: 3-phosphoinositide-dependent protein kinase-1
SREBP-1: Akt-sterol regulatory element-binding protein-1
Ang: Angiopoietin
ANG-1: Angiopoietin-1
ANGPTL: Angiopoietin-like proteins
BAD: Bcl-2-associated death promoter
eNOS: Endothelial nitric oxide synthase
EPC: Endothelial progenitor cells
ECM: Extracellular matrix
ERK: Extracellular signal-regulated kinases
FGFs: Fibroblast growth factors
GDM: Gestational diabetes
HbA1c: Glycosylated hemoglobin A1c
Grb-2: Growth factor receptor-bound protein 2
EGFL7: Growth factor-like domain
HOMA-IR: Homeostasis model assessment for insulin resistance
HUVEC: Human umbilical vein endothelial cell
IR: Insulin receptor
IGF-IR: Insulin-like growth factor receptors
IGF: Insulin-like growth factors
MAPK: Mitogen-activated protein kinase
MIRKO: Muscle-specific insulin receptor knockout
NRP: Neuropilins
NO: Nitric oxide
NOS: Nitric oxide synthase
PKC: Protein kinase C
PIP3: Phosphatidylinositol 3; 4; 5-trisphosphate
PI3K: Phosphoinositide 3-kinase
PLCγ1: Phospholipase Cγ1
PDGF-B: Platelet-derived growth factor B
PGI_2_: Prostacyclin
Rac-1: Ras-related C3 botulinum toxin substrate 1; SH2-containing proteins like PI3K
sflt-1: Soluble vascular endothelial growth factor receptor type 1 or soluble fms-like tyrosine kinase-1
Shb: Src homology 2 domain containing adaptor protein B
Scr: SRC proto-oncogene non-receptor tyrosine kinase
Tie: Tie receptor tyrosine kinases
TGF-β: Transforming growth factor-β
T1DM: Type 1 diabetes mellitus
T2DM: Type 2 diabetes mellitus
VEGF: Vascular endothelial growth factor
VEGFRs: VEGF receptors.
